# Supplementary material for: Metabolic basis for the evolution of a common pathogenic Pseudomonas aeruginosa variant
Source: eLife. 2022 May 3;11:e76555. doi: 10.7554/eLife.76555 (PMC9224983; doi:10.7554/eLife.76555)
Supplement: Supplementary file 3. — Samples are grouped according to CF status with non-CF donors denoted ‘HV’ with age group, gender, CFTR genotype, and percent forced expiratory volume in 1 s at the time of encounter listed. nd, not determined. [file elife-76555-supp3.docx]

| **Supplementary File 3. BAL Donor Metadata** | | | | | |
| --- | --- | --- | --- | --- | --- |
| *Sample* | *Group* | *Age* | *Sex* | *Genotype* | *%FEV_1_* |
| 1 | CF | 20-24 | Male | 508/508 | 113 |
| 2 | CF | 30-34 | Female | 508/508 | 92 |
| 3 | CF | 30-34 | Female | ClassI/Class III | 91 |
| 4 | CF | 25-29 | Male | 508/508 | 91 |
| 5 | CF | 25-29 | Male | 508/II–III–VI | 61 |
| 6 | CF | 30-34 | Male | 1B/? | 112 |
| 7 | CF | 18-21 | Male | 508/Class IB | 77 |
| 8 | CF | 30-34 | Male | 508/508 | 76 |
| 9 | CF | 25-29 | Male | 508/Class II | 59 |
| 10 | CF | 25-29 | Female | 508/508 | 63 |
| 11 | HV | 30-34 | Male | - | nd |
| 12 | HV | 25-29 | Female | - | nd |
| 13 | HV | 25-29 | Female | - | nd |
| 14 | HV | 25-29 | Female | - | nd |
| 15 | HV | 25-29 | Male | - | nd |
| 16 | HV | 25-29 | Female | - | nd |
| 17 | HV | 20-24 | Male | - | nd |
| 18 | HV | 30-34 | Female | - | nd |
| 19 | HV | 25-29 | Male | - | nd |
| 20 | HV | 25-29 | Female | - | nd |
| **As measured at time of encounter; nd, not determined* | | | | | |
