# Supplementary material for: Metabolic basis for the evolution of a common pathogenic Pseudomonas aeruginosa variant
Source: eLife. 2022 May 3;11:e76555. doi: 10.7554/eLife.76555 (PMC9224983; doi:10.7554/eLife.76555)
Supplement: Supplementary file 6. — List of strains and plasmids used in this study with internal lab strain identifier, short description of strain/use including gene name and gene number, and source listed. [file elife-76555-supp6.docx]

| **Supplementary File 6. Strains and plasmids used in this study** | | |  |
| --- | --- | --- | --- |
| Strain | Strain.ID | Description | Source |
| *P. aeruginosa* |  |  |  |
| PA14 WT | DH122 | Laboratory reference strain | (1) |
| PA14 *∆lasR* | DH164 | PA14 WT (DH122) with in-frame deletion of *lasR* (PA14_45960*)* | (2) |
| PAO-MW1qsc102 | DH161 | AHL-sensing bioreporter; PAO1 *lasIrhlI* mutant with Tn5-B22, which contains promoterless *lacZ* located within PA1896 (hypothetical protein) at chromosomal location of 2,067,716. Responsive to 3OC_12_-HSL but not C_4_-HSL. | (3, 4) |
| PAO-MW1qsc131 | DH162 | AHL-sensing bioreporter; PAO1 *lasIrhlI* mutant with Tn5-B22, which contains promoterless *lacZ,* under *phzC* promoter control. Responsive to either 3OC_12_-HSL or C_4_-HSL but requires both for full activation. | (3, 4) |
| PA14 WT *att::lacZ* | DH22 | PA14 WT with constitutive expression of *lacZ* | Roberto Kolter (5, 6) |
| PA14 WT | DH2455 | PA14 WT (strain background of kinase clean deletion mutants; parent of DH2456 - DH2518) | (7) |
| PA14 ∆*cheA* | DH2456 | PA14 WT (DH2455) with in-frame deletion in *cheA* (PA14_02250) | (7) |
| PA14 ∆*chpA* | DH2457 | PA14 WT (DH2455) with in-frame deletion in *chpA* (PA14_05390) | (7) |
| PA14 ∆*creC* | DH2458 | PA14 WT (DH2455) with in-frame deletion in *creC* (PA14_06070) | (7) |
| PA14 ∆*uhpB* | DH2459 | PA14 WT (DH2455) with in-frame deletion in *uhpB* (PA14_07820) | (7) |
| PA14 ∆*bfiS* | DH2460 | PA14 WT (DH2455) with in-frame deletion in *bfiS* (PA14_09680) | (7) |
| PA14 ∆*bphP* | DH2461 | PA14 WT (DH2455) with in-frame deletion in *bphP* (PA14_10700) | (7) |
| PA14 ∆PA14_10770 | DH2462 | PA14 WT (DH2455) with in-frame deletion in PA14_10770 | (7) |
| PA14 ∆PA14_11630 | DH2463 | PA14 WT (DH2455) with in-frame deletion in PA14_11630 | (7) |
| PA14 ∆*rocS1* | DH2464 | PA14 WT (DH2455) with in-frame deletion in *rocS1* (PA14_12820) | (7) |
| PA14 ∆*narX* | DH2465 | PA14 WT (DH2455) with in-frame deletion in *narX* (PA14_13740) | (7) |
| PA14 ∆*wspE* | DH2466 | PA14 WT (DH2455) with in-frame deletion in *wspE* (PA14_16470) | (7) |
| PA14 ∆PA14_19340 | DH2467 | PA14 WT (DH2455) with in-frame deletion in PA14_19340 | (7) |
| PA14 ∆*mxtR* | DH2468 | PA14 WT (DH2455) with in-frame deletion in *mxtR* (PA14_21700) | (7) |
| PA14 ∆*cpxS* | DH2469 | PA14 WT (DH2455) with in-frame deletion in *cpxS* (PA14_22730) | (7) |
| PA14 ∆*gtrS* | DH2470 | PA14 WT (DH2455) with in-frame deletion in *gtrS* (PA14_22960) | (7) |
| PA14 ∆PA14_24340 | DH2471 | PA14 WT (DH2455) with in-frame deletion in PA14_24340 | (7) |
| PA14 ∆*rocS2* | DH2472 | PA14 WT (DH2455) with in-frame deletion in *rocS2* (PA14_24720) | (7) |
| PA14 ∆PA14_26810 | DH2473 | PA14 WT (DH2455) with in-frame deletion in PA14_26810 | (7) |
| PA14 ∆*sagS* | DH2474 | PA14 WT (DH2455) with in-frame deletion in *sagS* (PA14_27550) | (7) |
| PA14 ∆*copS* | DH2475 | PA14 WT (DH2455) with in-frame deletion in *copS* (PA14_27800) | (7) |
| PA14 ∆*pfeS* | DH2476 | PA14 WT (DH2455) with in-frame deletion in *pfeS* (PA14_29360) | (7) |
| PA14 ∆*bqsS* | DH2477 | PA14 WT (DH2455) with in-frame deletion in *bqsS* (PA14_29740) | (7) |
| PA14 ∆PA14_30700 | DH2478 | PA14 WT (DH2455) with in-frame deletion in PA14_30700 | (7) |
| PA14 ∆PA14_30840 | DH2479 | PA14 WT (DH2455) with in-frame deletion in PA14_30840 | (7) |
| PA14 ∆*czcS* | DH2480 | PA14 WT (DH2455) with in-frame deletion in *czcS* (PA14_31950) | (7) |
| PA14 ∆PA14_32570 | DH2481 | PA14 WT (DH2455) with in-frame deletion in PA14_32570 | (7) |
| PA14 ∆PA14_36420 | DH2482 | PA14 WT (DH2455) with in-frame deletion in PA14_36420 | (7) |
| PA14 ∆*ercS* | DH2483 | PA14 WT (DH2455) with in-frame deletion in *ercS* (PA14_38740) | (7) |
| PA14 ∆e*xaD* | DH2484 | PA14 WT (DH2455) with in-frame deletion in *exaD* (PA14_38910) | (7) |
| PA14 ∆*ercS’* | DH2485 | PA14 WT (DH2455) with in-frame deletion in *ercS’* (PA14_38970) | (7) |
| PA14 ∆*parS* | DH2486 | PA14 WT (DH2455) with in-frame deletion in *parS* (PA14_41270) | (7) |
| PA14 ∆*kdpD* | DH2487 | PA14 WT (DH2455) with in-frame deletion in *kdpD* (PA14_43350) | (7) |
| PA14 ∆PA14_43670 | DH2488 | PA14 WT (DH2455) with in-frame deletion in PA14_43670 | (7) |
| PA14 ∆PA14_45590 | DH2489 | PA14 WT (DH2455) with in-frame deletion in PA14_45590 | (7) |
| PA14 ∆PA14_45870 | DH2490 | PA14 WT (DH2455) with in-frame deletion in PA14_45870 | (7) |
| PA14 ∆PA14_46370 | DH2491 | PA14 WT (DH2455) with in-frame deletion in PA14_46370 | (7) |
| PA14 ∆PA14_46980 | DH2492 | PA14 WT (DH2455) with in-frame deletion in PA14_46980 | (7) |
| PA14 ∆PA14_48160 | DH2493 | PA14 WT (DH2455) with in-frame deletion in PA14_48160 | (7) |
| PA14 ∆*phoQ* | DH2494 | PA14 WT (DH2455) with in-frame deletion in *phoQ* (PA14_49170) | (7) |
| PA14 ∆PA14_49420 | DH2495 | PA14 WT (DH2455) with in-frame deletion in PA14_49420 | (7) |
| PA14 ∆*fleS* | DH2496 | PA14 WT (DH2455) with in-frame deletion in *fleS* (PA14_50200) | (7) |
| PA14 ∆*pirS* | DH2497 | PA14 WT (DH2455) with in-frame deletion in *pirS* (PA14_52240) | (7) |
| PA14 ∆*gacS* | DH2498 | PA14 WT (DH2455) with in-frame deletion in *gacS* (PA14_52260) | (7) |
| PA14 ∆*tctE* | DH2499 | PA14 WT (DH2455) with in-frame deletion in *tctE* (PA14_54500) | (7) |
| PA14 ∆*pprA* | DH2500 | PA14 WT (DH2455) with in-frame deletion in *pprA* (PA14_55780) | (7) |
| PA14 ∆*colS* | DH2501 | PA14 WT (DH2455) with in-frame deletion in *colS* (PA14_56940) | (7) |
| PA14 ∆PA14_57170 | DH2502 | PA14 WT (DH2455) with in-frame deletion in PA14_57170 | (7) |
| PA14 ∆*roxS* | DH2503 | PA14 WT (DH2455) with in-frame deletion in *roxS* (PA14_58320) | (7) |
| PA14 ∆*rcsC* | DH2504 | PA14 WT (DH2455) with in-frame deletion in *rcsC* (PA14_59780) | (7) |
| PA14 ∆*pvrS* | DH2505 | PA14 WT (DH2455) with in-frame deletion in *pvrS* (PA14_59800) | (7) |
| PA14 ∆*pilS* | DH2506 | PA14 WT (DH2455) with in-frame deletion in *pilS* (PA14_60250) | (7) |
| PA14 ∆*cbrA* | DH2507 | PA14 WT (DH2455) with in-frame deletion in *cbrA* (PA14_62530) | (7) |
| PA14 ∆*pmrB* | DH2508 | PA14 WT (DH2455) with in-frame deletion in *pmrB* (PA14_63160) | (7) |
| PA14 ∆*retS* | DH2509 | PA14 WT (DH2455) with in-frame deletion in *retS* (PA14_64230) | (7) |
| PA14 ∆PA14_64580 | DH2510 | PA14 WT (DH2455) with in-frame deletion in PA14_64580 | (7) |
| PA14 ∆*aruS* | DH2511 | PA14 WT (DH2455) with in-frame deletion in *aruS* (PA14_65860) | (7) |
| PA14 ∆*ntrB* | DH2512 | PA14 WT (DH2455) with in-frame deletion in *ntrB* (PA14_67670 | (7) |
| PA14 ∆PA14_68230 | DH2513 | PA14 WT (DH2455) with in-frame deletion in PA14_68230 | (7) |
| PA14 ∆*amgS* | DH2514 | PA14 WT (DH2455) with in-frame deletion in *amgS/envZ* (PA14_68680) | (7) |
| PA14 ∆*algZ* | DH2515 | PA14 WT (DH2455) with in-frame deletion in *algZ* (PA14_69480) | (7) |
| PA14 ∆*phoR* | DH2516 | PA14 WT (DH2455) with in-frame deletion in *phoR* (PA14_70760) | (7) |
| PA14 ∆*kinB* | DH2517 | PA14 WT (DH2455) with in-frame deletion in *kinB* (PA14_72390) | (7) |
| PA14 ∆PA14_72740 | DH2518 | PA14 WT (DH2455) with in-frame deletion in PA14_72740 | (7) |
| PA14 ∆*rhlR* | DH2742 | PA14 WT (DH122) with in-frame deletion of *rhlR* | (8) |
| PA14 *∆anr* | DH2855 | PA14 WT (DH122) with in-frame deletion of *anr* (PA14_44490) | (9) |
| PA14 ∆*cbrB* | DH3920 | PA14 WT (DH122) with in-frame deletion of *cbrB* (PA14_62540) | This study |
| PA14 *cbrB*::Tn*M* | DH3921 | *cbrB MAR2xT7* transposon insertion mutant | (10, 11) |
| NC-AMT0101-1-2 | DH2417 | Chronic CF lung infection isolate with functional LasR allele | (12) |
| NC-AMT0101-1-2 ∆*cbrB* | DH3922 | CF clinical isolate (DH2417) with in-frame deletion of *cbrB* (PA14_62540) | This study |
| PA14 ∆*crc* | DH3737 | PA14 WT (DH122) with in-frame deletion of *crc* (PA14_70390) | This study |
| PA14 ∆*crc* + *crc* | DH3738 | PA14 ∆*crc* (DH3737) with complementation of *crc* (PA14_70390) at the native locus | This study |
| PA14 *∆lasR + lasR* | DH3549 | PA14 ∆*lasR* (DH164) with complementation of *lasR* (PA14_45960) at the native locus | (13) |
| PA14 ∆*cbrB* + pMQ70 *cbrB* | DH3923 | PA14 ∆*cbrB* (DH3920) expressing arabinose inducible pMQ70 *cbrB* expression vector | This study |
| PA14 *∆lasR*∆*cbrB* | DH3924 | PA14 ∆*lasR* (DH164) with in-frame deletion of *cbrB* (PA14_62540) | This study |
| PA14 *∆lasR*∆*cbrB* + *cbrB* | DH3925 | PA14 ∆*lasR*∆*cbrB* (DH3924) with complementation of *cbrB* (PA14_62540) at the native locus | This study |
| PA14 *∆lasR*∆*cbrB∆crc* | DH3926 | PA14 ∆*lasR*∆*cbrB* (DH3924) with in-frame deletion of *crc* (PA14_70390) | This study |
| PA14 *∆lasR∆crc* | DH3927 | PA14 ∆*lasR* (DH164) with in-frame deletion of *crc* (PA14_70390) | This study |
| PA14 *∆lasR∆crc* + *crc* | DH3928 | PA14 ∆*lasR*∆*crc* (DH3927) with complementation of *crc* (PA14_70390) at the native locus | This study |
| PA14 + pMQ72 EV | DH3929 | PA14 WT (DH122) expressing pMQ72 empty vector | This study |
| PA14 + pMQ70 *cbrB* | DH3930 | PA14 WT (DH122) expressing arabinose inducible pMQ70 *cbrB* expression vector | This study |
| PA14 + pMQ72 *crcZ* | DH3932 | PA14 WT (DH122) expressing arabinose inducible pMQ72 *crcZ* expression vector | This study |
| *E. coli* |  |  |  |
| S17 λpir | DH71 | Used as a conjugation partner for introducing pMQ30 plasmids. |  |
| DH5a | DH51 | Used to store/replicate plasmids. | Invitrogen |
| Plasmids |  |  |  |
| pMQ30 EV | DH962 | Allelic replacement vector, Gm^R^ | (14) |
| pMQ72 EV | DH3773 | Arabinose inducible expression vector, Gm^R^ | (14) |
| pMQ70 EV | DH1682 | Arabinose inducible expression vector, Amp^R^ | (14) |
| pMQ30*_rhlR_*KO | DH2943 | *rhlR* (PA14_19120) in-frame deletion construct; Gm^R^ | This study |
| pMQ30*_cbrB_*KO | DH3512 | *cbrB* (PA14_62540) in-frame deletion construct; Gm^R^ | This study |
| pMQ30*_crc_*KO | DH3692 | *crc* (PA14_70390) in-frame deletion construct; Gm^R^ | This study |
| pMQ30*_cbrB_*KON | DH3467 | *cbrB* (PA14_62540) native locus complementation construct; Gm^R^ | This study |
| pMQ30*_crc_*KON | DH3511 | *crc* (PA14_70390) native locus complementation construct; Gm^R^ | This study |
| pMQ72*_crcZ* | DH3931 | Vector for arabinose-inducible gene expression of *crcZ*; Gm^R^ | This study |
| pMQ70*_cbrB* | DH3691 | Vector for arabinose-inducible gene expression of *cbrB* (PA14_62540); Carb^R^ | This study |
|  |  |  |  |
|  |  |  |  |
|  |  |  |  |

**References**

1. Rahme LG, Stevens EJ, Wolfort SF, Shao J, Tompkins RG, Ausubel FM. 1995. Common virulence factors for bacterial pathogenicity in plants and animals. Science 268:1899-902.

2. Hogan DA, Vik A, Kolter R. 2004. A *Pseudomonas aeruginosa* quorum-sensing molecule influences *Candida albicans* morphology. Mol Microbiol 54:1212-23.

3. Whiteley M, Lee KM, Greenberg EP. 1999. Identification of genes controlled by quorum sensing in *Pseudomonas aeruginosa*. Proc Natl Acad Sci U S A 96:13904-9.

4. Whiteley M, Greenberg EP. 2001. Promoter specificity elements in *Pseudomonas aeruginosa* quorum-sensing-controlled genes. J Bacteriol 183:5529-5534.

5. Wang Z, Xiong G, Lutz F. 1995. Site-specific integration of the phage phi CTX genome into the *Pseudomonas aeruginosa* chromosome: characterization of the functional integrase gene located close to and upstream of *attP*. Mol Gen Genet 246:72-9.

6. Choi KH, Schweizer HP. 2006. mini-Tn7 insertion in bacteria with single *att*Tn7 sites: example *Pseudomonas aeruginosa*. Nat Protoc 1:153-61.

7. Wang BX, Cady KC, Oyarce GC, Ribbeck K, Laub MT. 2021. Two-component signaling systems regulate diverse virulence-associated traits in *Pseudomonas aeruginosa*. Appl Environ Microbiol 87.

8. Harty CE, Martins D, Doing G, Mould DL, Clay ME, Occhipinti P, Nguyen D, Hogan DA. 2019. Ethanol stimulates trehalose production through a SpoT-DksA-AlgU dependent pathway in *Pseudomonas aeruginosa*. Journal of Bacteriology doi:10.1128/jb.00794-18:JB.00794-18.

9. Crocker AW, Harty CE, Hammond JH, Willger SD, Salazar P, Botelho NJ, Jacobs NJ, Hogan DA. 2019. *Pseudomonas aeruginosa* ethanol oxidation by AdhA in low-oxygen environments. J Bacteriol 201.

10. Feinbaum RL, Urbach JM, Liberati NT, Djonovic S, Adonizio A, Carvunis AR, Ausubel FM. 2012. Genome-wide identification of *Pseudomonas aeruginosa* virulence-related genes using a *Caenorhabditis elegans* infection model. PLoS Pathog 8:e1002813.

11. Liberati NT, Urbach JM, Miyata S, Lee DG, Drenkard E, Wu G, Villanueva J, Wei T, Ausubel FM. 2006. An ordered, nonredundant library of *Pseudomonas aeruginosa* strain PA14 transposon insertion mutants. Proceedings of the National Academy of Sciences of the United States of America 103:2833-2838.

12. Smith EE, Buckley DG, Wu Z, Saenphimmachak C, Hoffman LR, D'Argenio DA, Miller SI, Ramsey BW, Speert DP, Moskowitz SM, Burns JL, Kaul R, Olson MV. 2006. Genetic adaptation by *Pseudomonas aeruginosa* to the airways of cystic fibrosis patients. Proc Natl Acad Sci U S A 103:8487-92.

13. Clay ME, Hammond JH, Zhong F, Chen X, Kowalski CH, Lee AJ, Porter MS, Hampton TH, Greene CS, Pletneva EV, Hogan DA. 2020. *Pseudomonas aeruginosa lasR* mutant fitness in microoxia is supported by an Anr-regulated oxygen-binding hemerythrin. Proceedings of the National Academy of Sciences 117:3167-3173.

14. Shanks RM, Caiazza NC, Hinsa SM, Toutain CM, O'Toole GA. 2006. *Saccharomyces cerevisiae*-based molecular tool kit for manipulation of genes from gram-negative bacteria. Appl Environ Microbiol 72:5027-36.
