# Supplementary material for: Metabolic basis for the evolution of a common pathogenic Pseudomonas aeruginosa variant
Source: eLife. 2022 May 3;11:e76555. doi: 10.7554/eLife.76555 (PMC9224983; doi:10.7554/eLife.76555)
Supplement: Supplementary file 7. — Description of the terms for lag, growth, and death for both wild type strain PA14 and derived lasR mutants, and estimates of the number of lasR mutants in the initial population. [file elife-76555-supp7.docx]

**Model of evolution of *lasR* mutants through serial passages**

To model the evolution of *lasR* loss-of-function strains in the serial passage experiments, we consider a simple competition for resources between wild type and *lasR* mutants, without any further interactions between the two genotypes. We model the growth of each subpopulation through each passage in three phases: lag, growth, and death. We base our model on standard logistic growth equations, with parameters for each genotype obtained from growth curves for each genotype measured experimentally. At the end of each passage, the final mixed population is sampled at 1/200 for the next passage.

Since the time that it takes for *lasR* mutants to take over the population is reproducible, the appearance of *lasR* mutations is not a rare event, and mutants are likely to already be present in the initial population at the beginning of the experiment. This hypothesis is supported by the presence of multiple different *lasR* mutations in the evolved population. We estimate this initial population of *lasR* mutants to be in the order of tens of copies in 5 mL of saturated culture. This is the approximate number of mutations expected to happen within the *lasR* open reading frame in each turnover of that population $n_{0}=\mu_{PA}l_{lasR}d_{PA}v/g_{PA}\approx60$, where $\mu_{PA}=0.52 \times{10}^{-3}$ is the genome-wide mutation rate per generation of *P. aeruginosa*^1^, $l_{lasR}=720 bp$ is the length of the *lasR* gene, $g_{PA}\approx6Mbp$ is the length of the *P. aeruginosa* genome^2^, $d_{PA}=2\times{10}^{8}cells/ml$ is the density of a saturated culture and $v=5ml$ is the volume of the culture. Therefore, *lasR* mutants are expected to escape genetic drift and establish a robust subpopulation early in the evolution experiment.

*Lag phase*: we assume that *lasR* mutants have a shorter lag phase than wild-type cells, initiating growth earlier. The population of mutants then grows exponentially without competition, until wild-type cells also exit the lag phase. Assuming *lasR* mutants grow at their maximum rate $\lambda_{mut}$ during this period, the mutant population $n_{mut}$ is increased by a factor of $e^{\lambda_{mut}\left( L_{wt}-L_{mut} \right)}$ by the time wild-type cells exit the lag phase, while the wild-type population $n_{wt}$ remains constant. $L_{mut}$ and $L_{wt}$ are the durations of the respective lag phases.

*Growth phase*: we model the growth of wild type cells and *lasR* mutants using coupled differential equations for logistic growth while competing for resources. With $K$ denoting the carrying capacity of each genotype, we have:

$$\dot{n_{wt}}=\lambda_{wt}n_{wt}\left( 1-\frac{n_{wt}+\left( \lambda_{mut}/\lambda_{wt} \right) n_{mut}}{K_{wt}} \right)$$

$$\dot{n_{mut}}=\lambda_{mut} n_{mut}\left( 1-\frac{n_{mut}+\left( \lambda_{wt}/\lambda_{mut} \right)n_{wt}}{K_{mut}} \right)$$

We modify these equations slightly to ensure that $\dot{n_{mut}}$, $\dot{n_{wt}}>0$, reflecting the fact that these subpopulations do not decline when nutrients become scarce during the growth phase, but simply stop growing. These equations account for distinct growth rates $\lambda$ and carrying capacities $K$ for wild type and mutant cells, which are determined experimentally. The coupling of the equations implements direct competition for resources, with the abundance of one genotype being limited by the abundance of the other.

*Death phase*: after the culture reaches saturation, it enters a death phase where the population declines slowly. We model this process through an exponential decay, with a decay rate $\sigma$ that encompasses both cell death and a possible offset due to residual growth on nutrients released by lysed cells. During this period, the wild type and mutant populations are decreased by factors of $e^{{-\sigma}_{wt}T_{D}}$ and $e^{{-\sigma}_{mut}T_{D}}$ respectively, where $T_{D}$ is the duration of the death phase.

We simulate this system iteratively for each passage, with the starting population being a 1/200 sample of the final population of the previous iteration. We use MatLab ODE solver *ode113* to integrate the system of equations over time.

Model Parameters Table

|  |  | WT | $\Delta$lasR |
| --- | --- | --- | --- |
| Lag (hour) | $L$ | 1 | 0 |
| Growth rate (1/hour) | $\lambda$ | 1.9 | 1.615 |
| Carrying capacity | $K$ | 0.8 | 1.2 |
| Killing Rate (1/hour) | $\sigma$ | 0.07 | 0.07 |

References

1. Dettman, J. R., Sztepanacz, J. L., & Kassen, R. (2016). The properties of spontaneous mutations in the opportunistic pathogen Pseudomonas aeruginosa. *BMC genomics*, *17*, 27. <https://doi.org/10.1186/s12864-015-2244-3>

2. Subedi, D., Vijay, A.K., Kohli, G.S. *et al.* Comparative genomics of clinical strains of *Pseudomonas aeruginosa* strains isolated from different geographic sites. *Sci Rep* **8,**15668 (2018). <https://doi.org/10.1038/s41598-018-34020-7>
